# Comprehensive genomic and phenotypic metal resistance profile of *Pseudomonas putida* strain S13.1.2 isolated from a vineyard soil

**DOI:** 10.1186/s13568-016-0269-x

**Published:** 2016-10-12

**Authors:** Teik Min Chong, Wai-Fong Yin, Jian-Woon Chen, Samuel Mondy, Catherine Grandclément, Denis Faure, Yves Dessaux, Kok-Gan Chan

**Affiliations:** 1 Division of Genetics and Molecular Biology, Institute of Biological Sciences, Faculty of Science, University of Malaya, 50603 Kuala Lumpur, Malaysia; 2 Institute for Integrative Biology of the Cell (I2BC), CEA, CNRS, Université Paris-Sud, Université Paris-Saclay, 91198 Gif-sur-Yvette, France

**Keywords:** *Pseudomonas putida*, Single-molecule real-time (SMRT) sequencing, Vineyard soil, Copper resistance, Heavy metal resistance

## Abstract

Trace metals are required in many cellular processes in bacteria but also induce toxic effects to cells when present in excess. As such, various forms of adaptive responses towards extracellular trace metal ions are essential for the survival and fitness of bacteria in their environment. A soil *Pseudomonas putida*, strain S13.1.2 has been isolated from French vineyard soil samples, and shown to confer resistance to copper ions. Further investigation revealed a high capacity to tolerate elevated concentrations of various heavy metals including nickel, cobalt, cadmium, zinc and arsenic. The complete genome analysis was conducted using single-molecule real-time (SMRT) sequencing and the genome consisted in a single chromosome at the size of 6.6 Mb. Presence of operons and gene clusters such as *cop*, *cus*, *czc*, *nik,* and *asc* systems were detected and accounted for the observed resistance phenotypes. The unique features in terms of specificity and arrangements of some genetic determinants were also highlighted in the study. Our findings has provided insights into the adaptation of this strain to accumulation and persistence of copper and other heavy metals in vineyard soil environment.

**Electronic supplementary material:**

The online version of this article (doi:10.1186/s13568-016-0269-x) contains supplementary material, which is available to authorized users.

## Introduction

Natural or anthropogenic accumulation of heavy metals in the environment could be tenacious and exhibit toxicity towards living organisms. Though minute amounts of these metals (e.g. Cu, Ni, Zn) are required in several cellular processes, excessive heavy metal ion concentrations can also exert deleterious effects by catalyzing oxidation of lipid membrane, damaging nucleic acids, and producing free radicals (Cánovas et al. 2003; Thounaojam et al. 2012; Wang et al. 2004). Consequently, presence of metal stress can alter the microbial composition and functional diversity of microbial communities in relation with a decreased biomass of metal-sensitive microbes (Bardgett et al. 1994; Joynt et al. 2006; Kandeler et al. 1996). As such, it is likely that tolerant soil inhabitants acquired resistance systems towards a range of heavy metal found in soil environments, to maintain their fitness and survival capabilities. These resistance determinants, comprising operons or gene clusters, have been evidenced either in the chromosomes or plasmids of numerous Gram-positive and Gram-negative bacteria (García-Domínguez et al. 2000; Karelová et al. 2011; Wang and Chen 2006).

Among the wide diversity of resistance mechanisms found in prokaryotes, the extrusion of cations, driven by transmembrane efflux pumps are well described. This includes, for instance, resistance-nodulation-cell division (RND) superfamily proteins that form complexes with (i) outer membrane factors (OMF) or (ii) membrane fusion proteins. These two complexes are involved in transport of heavy metal ions from cytoplasm to periplasm, or across outer membrane from periplasm to outside of the cell (Nies 2003). This phenomenon is exemplified by the CzcCBA system that mediates an efflux of Co^2+^, Zn^2+^ and Cd^2+^ ions (Nies 2000). Another family of heavy metal transport protein, namely P-type ATPases, are involved in both import of inorganic cations to cytoplasm from periplasm or outside of the cell, and export of these ions from/to cytoplasm in a reversed manner. Such mechanism requires ATP hydrolysis. The reported heavy metal substrates for this system include Cu^2+^, Ag^+^, Cd^2+^ and Zn^2+^ (Fagan and Saier 1994). Aside from ion transport, detoxification of heavy metal ions is also essential for bacteria. For instance, reduction of the pentavalent arsenate [As(V)] ion to its trivalent arsenite [As(III)] counterpart by the arsenate reductase ArsC enables the detoxification and efflux of As(III) through the membrane pump protein ArsB (Cai et al. 1998; Carlin et al. 1995). In addition, these systems are often regulated in response to the presence of metal ions that in turn activates transcription of subsequent resistance determinants. Examples include the regulator ArsR that induces the expression of the *ars* arsenite/antimonite resistance operon upon exposure to these ions (Sato and Kobayashi 1998), or CzcD, a cation diffusion facilitator protein that partially regulates the expression of the CzcCBA system (Nies 1992).

Copper-containing pesticides have long been used, primarily for the control of plant pathogens and associated diseases especially in vineyards (Cooksey 1993). Indeed, *Vitis vinifera* is a non-rotating grape crop that has been routinely treated with copper sulphate over the years in order to control fungal diseases (Andreazza et al. 2010). Consequently, such anthropogenic accumulation of copper selects for the prevalence of copper resistant microorganisms that carry the genetic determinants involved in acquisition, efflux, sequestration or cellular distribution of copper (Andreazza et al. 2010; Cervantes and Gutierrez-Corona 1994; Munson et al. 2000). This feature has sparked interest to identify the microbiota inhabiting these soils and the abilities to tolerate elevated amount of copper ions as well as that of other heavy metal ions. Here, we described the heavy metal resistance phenotypes of *Pseudomonas putida* strain S13.1.2 isolated from a vineyard in France and the profile of possible genetic determinants responsible for metal resistance was also reported in the study.

## Materials and methods

### Sampling and bacteria isolation

In this study, strain S13.1.2 was isolated from a vineyard soil sample obtained in Riquewihr, France. Collection of soil sample was performed at subsurface level (to the depth of 5 cm). Isolation of this strain was performed using KG medium supplemented with 500 mg/L *N*-heptanoyl-homoserine lactone as sole carbon and nitrogen source (Chan et al. 2009). Next, the strain was identified using both 16S rRNA gene sequencing analysis using 27F (5′-AGAGTTTGATCMTGGCTCAG-3′) (Lane 1991) and 1525R (5′-AAGGAGGTGWTCCARCC-3′) (Dewhirst et al. 2000) primers pair followed by Microflex MALDI-TOF (Bruker Daltonik GmbH, Leipzig, Germany) bench-top mass spectrometer (Chen et al. 2013). The pure culture was routinely maintained on LB (Luria–Bertani, Merck, Whitehouse Station, NJ, USA) agar at 28 °C or cultivated in LB broth agitated at 220 rpm.

### Copper tolerance assay

Maximum tolerable concentration of copper sulphate salt (CuSO_4_) for the growth of *P*. *putida* S13.1.2 was determined. To do so, 1 µL of an overnight culture was inoculated into 200 µL of LB broth supplemented with different concentrations of CuSO_4_ ranging from 0 to 5 mM and placed into a 96-wells microtitre plate. The growth curves of the strain were monitored at OD_600_ with measurements taken at the interval of 30 min for 24 h using a Tecan Infinite M200 luminometer (Tecan, Mannerdorf, Switzerland).

### Biolog phenotype microarray analysis

The phenotypes associated with the heavy metal resistance of strain S13.1.2 were assessed using biolog phenotype microarray analysis. The overnight cultured bacterial colonies were inoculated into Biolog IF-0a GN/GP Base medium to reach 85 % turbidity followed by 1:200 dilution aliquoted into IF-10b medium supplemented with Dye Mix A as indicated by the manufacturer instructions. The mixture were then added into wells of Biolog Microplates PM13B, PM14A, PM15B, PM16A and PM18C containing substrates of various heavy metal salts. The incubation and growth of inocula were monitored for 96 h with readings taken at 15 min intervals. Growth of bacteria in the presence of heavy metals causes reduction of the dye, resulting in purple colour formation. The kinetic information were recorded and quantified (Bochner et al. 2001) using OmniLog OL_FM_12 kinetic software (Biolog, USA) for data analysis and export.

### Genomic DNA preparation and complete genome sequencing

Bacterial genomic DNA was extracted using MasterPure™ Complete DNA and RNA Purification Kit (Epicentre, Illumina Inc., Madison, Wisconsin) according to the manufacturer instructions. The purity of DNA was examined using a NanoDrop spectrophotometer (Thermo Scientific, Waltham, MA, USA) and agarose gel electrophoresis. DNA quantification was carried out with a Qubit^®^ 2.0 fluorometer (dsDNA Broad Range Assay Kit, Invitrogen, Carlsbad, CA, USA). The genome of strain S13.1.2 was sequenced using a PacBio RSII platform. Prior to sequencing, the preparation of the DNA library was performed using a Template Preparation Kit (Pacific Biosciences, Inc., CA) with fragments size targeted at 10 kb. For completion sequencing of the genome, sequence collection was carried out in 7 SMRT cells using P4/C2 chemistry for 180 min for each cell.

### Genome assembly and annotation

The acquired sequence reads were subjected to quality filtering and de novo assembly using the hierarchical genome-assembly process (HGAP) version 3.0 module available from the Pacific Biosciences’s SMRT portal (Chin et al. 2013). Circularization of the genome was verified using Gepard version 1.30 (Krumsiek et al. 2007) followed by curation using to Amos (http://www.amos.sourceforge.net/wiki/index.php/ToAmos) and minimus2 (http://www.amos.sourceforge.net/wiki/index.php/Minimus2) programs. The genes in the assembled sequences were then predicted and annotated using Rapid Prokaryotic Genome Annotation (Prokka) (Seemann 2014). Open reading frames attributed to heavy metal resistance were additionally validated by sequence comparison with NCBI-NR using BLAST program. The genome of *P. putida* S13.1.2 was also deposited at the integrated microbial genome (IMG) platform (http://www.img.jgi.doe.gov/) for additional gene prediction and manual functional annotation (Markowitz et al. 2012).

### Accession number

The complete genome sequence has been also deposited at NCBI under accession number CP010979. The *P*. *putida* strain S13.1.2 is available at DSMZ (accession no. DSM 102034).

## Results

### Isolation and characterization of strain S13.1.2

Sequence comparison with GenBank databases using the BLASTN followed by phylogenetic analysis revealed the closest identified relative to *P*. *putida* NBRC 14164 sharing 99 % of sequence identity (Additional file 1: Figure S1). Additional identification using MALDI–TOF–MS analysis also showed coherent identification of *P*. *putida* as the closest relative with matching score constantly above the values of 1.9.

### Copper resistance and other heavy metal resistance traits of strain S13.1.2

**Fig. 1:**
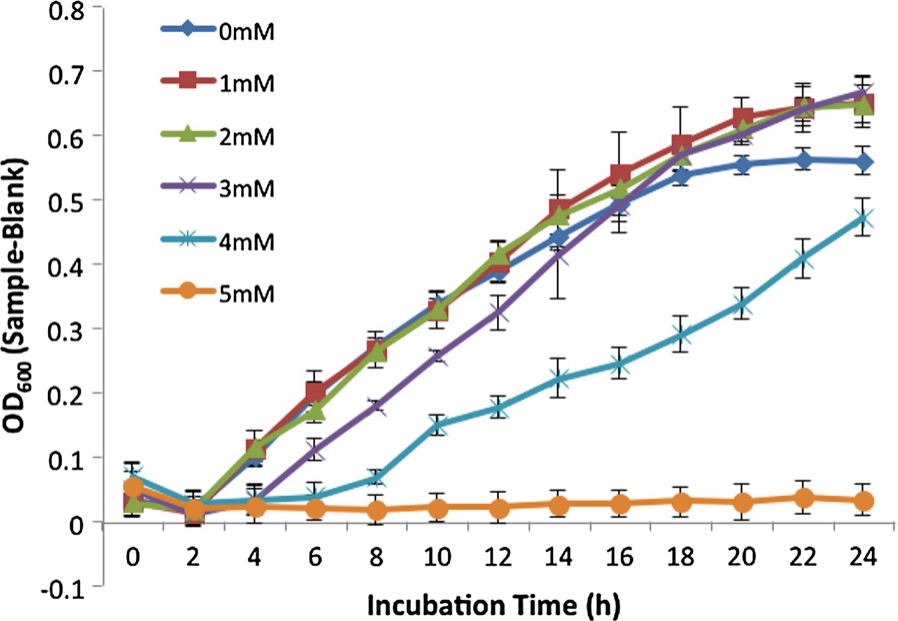
The growth curve of strain S13.1.2 in various copper sulphate salt (CuSO_4_) concentrations monitored for 24 h. The *graphs* represent mean results from (OD_600_—blank) values of triplicate cultures with *error bars* signifying standard deviations (n = 3). Growth was observed at up to 4 mM of CuSO_4_ supplemented into the culture medium

Growth of *P*. *putida* S13.1.2 in the presence of various CuSO_4_ concentrations in LB medium was observed in media supplemented with up to 3.5 mM of the copper salt. Though with a slightly hindered growth, strain S13.1.2 was also able to grow in 4 mM of CuSO_4_ (Fig. 1). The capability of S13.1.2 to tolerate different heavy metals was determined via biolog phenotype microarray analysis. After the incubation time of 96 h, complete growth curves were observed in the presence of almost all tested heavy metal salts (Table 1). Such observations prompted us to search for genetic determinants involved in copper resistant ability of this strain.

**Table 1:** Growth of *Pseudomonas* strain S13.1.2 in presence of various heavy metal salts from PM13B, PM14A, PM15B, PM16A and PM18C

Biolog microplates	Heavy metal salt	Growth observed
PM13B	NiCl_2_	+++
	K_2_CrO_4_	++
	CsCl	++++
	CoCl_2_	+++
	MnCl_2_	++
	CuCl_2_	++++
PM14A	CdCl_2_	++
	Na_3_AsO_4_	++++
PM15B	ZnCl_2_	++++
PM16A	NaSeO_3_	++
	CrCl_3_	++++
PM18C	NaAsO_2_	+++
	SbCl_3_	−

Heavy metal resistance profile is defined by bacterial growth measured after 96 h in phenotype microarray microplates wells containing four different concentrations of each heavy metal salts. The extent of growth observed were indicated as ++++ (full growth), +++ (strong), ++ (moderate), + (weak) and −(sensitive) signs

### Genome properties

HGAP assembly and circularization of the genome of *P*. *putida* S13.1.2 has yielded a single contig with a final assembled genome size of 6621,848 bp and a sequencing depth of 163.56-fold. This led us to conclude that the genome of *P. putida* S13.1.2 consisted in one circular chromosome with a 62.34 % G + C content (Additional file 1: Table S2). As displayed in the IMG/ER gene prediction and annotation, 5979 genes were predicted, 5814 (97.24 %) of which being protein-coding genes. An amount of 4923 genes among the protein coding genes were assigned with putative functions with the remaining 891 genes predicted as hypothetical proteins. In addition, 165 RNA genes were predicted with 22 genes assigned as rRNA and 75 genes as tRNA. The properties and the statistics of the genome are summarized in Table 2. The genomic features were also presented in IMG/ER database (https://www.img.jgi.doe.gov/cgi-bin/mer/main.cgi?section=TaxonDetail&page=taxonDetail&taxon_oid=2609459728).

**Table 2:** Nucleotide content and gene count levels of the genome predicted in IMG/ER

Attribute	Genome (total)	
	Value	% of total^a^
Size (bp)	6,621,848	100
G + C content (bp)	4,128,086	62.34
Coding region (bp)	5,922,241	89.43
Total genes	5979	100
RNA genes	165	2.76
Protein-coding genes	5814	97.24
Genes in paralog clusters	4841	80.97
Genes assigned to COGs	4343	72.64
Genes with signal peptides	643	10.75
Genes with transmembrane helices	1324	22.14
Paralogous groups	0	–
Pseudogenes	0	–

^a^ The total is based on either the size of the genome in base pairs or the total number of protein coding genes in the annotated genome

### In silico identification of copper resistance genes

**Fig. 2:**
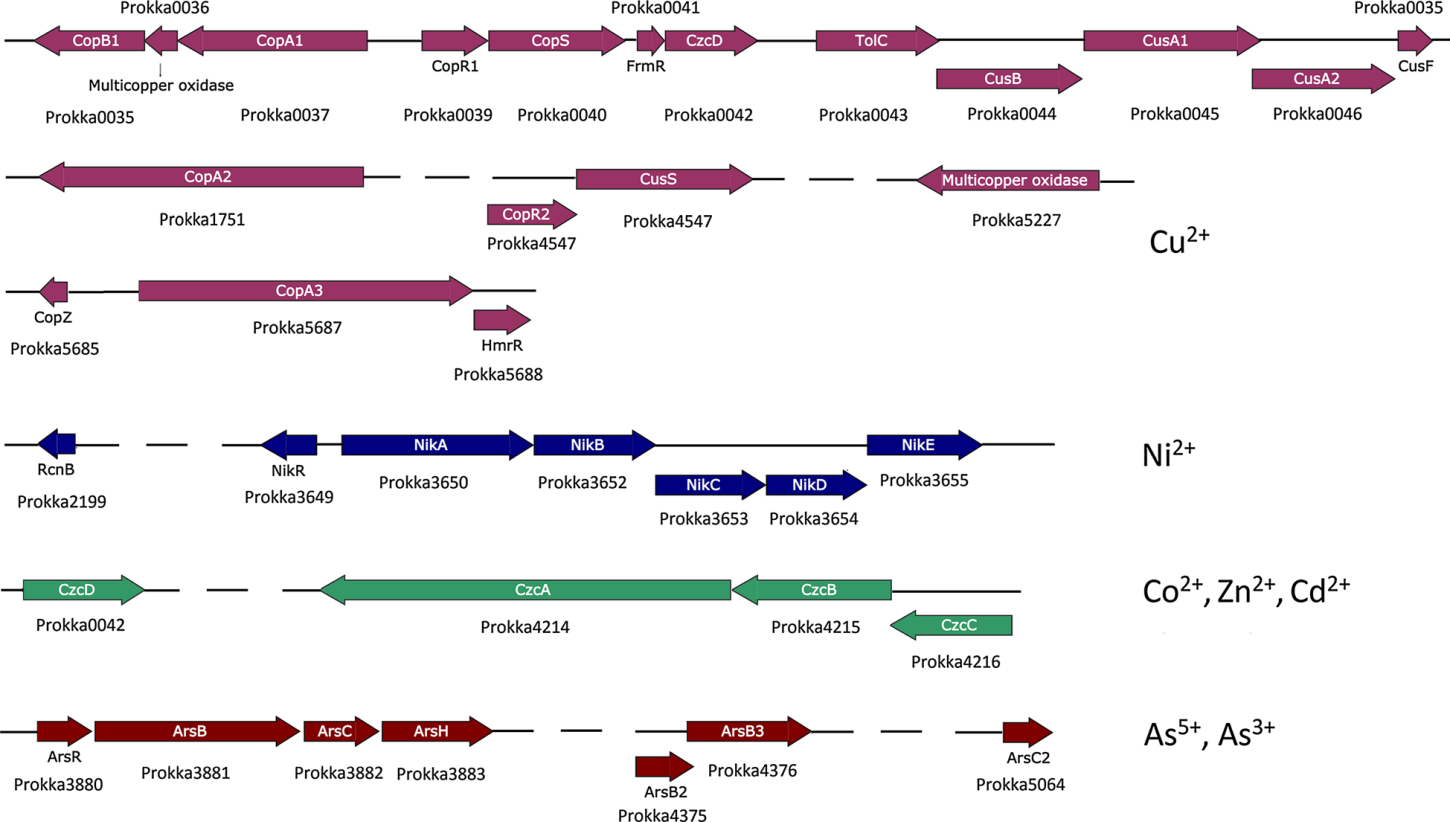
Orientation and product of putative gene clusters and operons involved in heavy metal resistance determinants annotated using PROKKA. Annotated genes attributed to copper, nickel and arsenic are displayed in *purple*, *blue* and *brown*, respectively whereas components of *czc* operon were presented as cyan

A total of 18 putative copper resistance genes, orthologous to genes associated with copper homeostasis and copper transport, were identified at six locations of the *P*. *putida* strain S13.1.2 genome (Fig. 2; Additional file 1: Table S1). For transport of copper ions, the genetic determinants *copA2* and *copA3* encoding copper-importing P-type ATPase A and copper-exporting P-type ATPase A, respectively, were identified. Besides, a gene cluster composed of *copB1*, *mco*, and *copA1* that encode copper resistance protein B, multicopper oxidase and copper resistance protein A, respectively was also present.

Aside from *cop* genes, a gene cluster consisting in *cusA1*, *cusA2* and *cusB* that coded cation efflux system proteins and *cusF* that coded a copper binding periplasmic protein were present. All these genes were components of a putative operon (*cusCFBA*) that determined an efflux pump for copper and silver ions. However genes encoding the CusC outer membrane channel component, belonging to OMF family was absent (Kulathila et al. 2011). Instead, the outer membrane efflux protein located upstream of CusB gene showed higher similarity towards TolC, another OMF that is described as part of the AcrAB-TolC multidrug efflux pump system (Balakrishnan et al. 2001; Rensing and Grass 2003). Although TolC was shown not to restore metal resistance in CusC knockouts in *Escherichia coli* (Franke et al. 2003), such a combination might represent a new copper transport mechanism in *P*. *putida*.

### Genetic determinants for other heavy metal resistance

Following the phenotype microarray analysis that revealed resistance towards various heavy metals, a series of determinants were also identified in the genome of S13.1.2 (Fig. 2; Additional file 1: Table S1). The genes that likely contributed to resistance traits included the *nikRABCDE* putative operon that mediated uptake of nickel ions, in which *nikR* negatively regulates the expression when in excess of nickel ions (Chivers and Sauer 2000; Navarro et al. 1993).

Aside, a gene cluster that grouped *czc* cobalt-zinc-cadmium resistance determinants was found. CzcC, CzcB and CzcA heavy metal efflux proteins are involved in the efflux of heavy metals ions such as Co^2+^, Cd^2+^ and Zn^2+^ (Nies 1992, 2000). The deduced CzcA, CzcB and CzcC proteins of *P*. *putida* strain S13.1.2 belonged to the family of CzcA, CzcB and CzcC heavy metal RND efflux proteins in the *P*. *putida* group. A *czcD* gene involved in the repression of *czc* system was also present in the genome (Anton et al. 1999; van der Lelie et al. 1997).

Strain S13.1.2 also appeared to be highly resistant towards As(V) salt (microplate PM14A) and moderately towards As(III) salt (microplate PM18C). The genome sequence revealed the presence of an arsenate resistance operon (*ars*) that consisted in *arsR*, *arsB1*, *arsC1* and *arsH* determinants, together with *arsB2*, *arsB3 and arsC2* genes at different locations in the genome.

## Discussion

The genome sequence of *P. putida* strain S13.1.2 isolated from a vineyard soil revealed that it contained a series of *cop* and *cus* genes associated with noticeable in vivo resistance to copper in this study. Identification of *cop* genes strongly suggested the occurrence of a resistance mechanism based on protein-mediated sequestration and cellular accumulation of the copper ions in the cell (Cooksey 1993). Notably, it is also highly possible that the transport of copper is further facilitated by *cus* operon. Activation of this transport mechanism is likely mediated by the *copR1* and *copS* genes located upstream of the *cus* gene cluster that encodes the transcriptional activator and sensor kinase. In *Pseudomona syringae*, this pair forms a two-component regulatory system whereby phosphorylation of CopR by CopS induces the expression of the copper resistance operon (Mills et al. 1993). Another set of transcriptional activator and sensor kinase genes (*copR2 and cusS*) was also found in the genome. However sequence alignments between both gene pairs showed a low identity (52.9 %) and similarity (66.1 %) at the protein level that suggested their involvement in dissimilar copper resistance mechanisms.

The copper resistant trait of this strain could also be discussed in the light of a reported result where another copper resistant *Pseudomonas* strain was also isolated from the same environment, namely *P*. *mendocina* strain S5.2. The genome of *P*. *mendocina* strain S5.2 also harbors genes that encode proteins predicted to be involved in heavy-metal transport/detoxification and heavy-metal resistance such as heavy-metal-translocating P-type ATPases, known for their role in heavy metal ion homeostasis and biotolerance of heavy-metal ions (e.g. Cu^2+^, Cd^2+^, Zn^2+^, and Ag^+^) (Chong et al. 2012). Furthermore, these bacteria are part of the grapevine endophytic microbiome and are frequently detected in vineyard soils (Salomon et al. 2014; West et al. 2010). In addition to this, pseudomonads often exhibit elevated copper resistance (Andreazza et al. 2012).

As concentrations up to 1000 mg copper sulphate per kg soil can still be found in some places (Flores-Vélez et al. 1996) and surface layer of soil poses the highest level of copper as reported by Angelova et al. (1999) isolation of *Pseudomonas* strains at this depth (5 cm) hereby corresponds to the copper resistance traits. Therefore, anthropogenic accumulation of copper likely explains the prevalence of *Pseudomonas* that could support the growth of plants treated with copper sulphate over the years in order to control fungal diseases.

Furthermore the presence of *nik*, *czc* and *ars* operons in the genome that corresponds to resistance phenotypes towards their respective heavy metals also drove speculation on the tenacity of other vineyard soil chemistries in this study. Of note, vineyards have also been treated with sodium arsenate till the end of the last century, and vineyard posts in several parts of the world have been treated with a mixture of copper-chromium-arsenic salts. As a consequence, chromium and arsenic salts in vineyards soils and surrounding fields may remain at detectable concentrations [e.g. (Robinson et al. 2006)]. Hence identification of *ars* operon and resistance in this strain indicates the persistence of arsenic in this vineyard soil environment.

The *ars* operons are quite diverse (Branco et al. 2008) in pseudomonads such as *P*. *aeruginosa* (Cai et al. 1998) and *P*. *putida* (Fernández et al. 2014). In response to the presence of arsenite, the transcriptional repressor ArsR bound to the cognate promoter is released, followed by the subsequent induction of the *ars* gene expression (Busenlehner et al. 2003; Murphy and Saltikov 2009). These include the transmembrane efflux pump ArsB that extrudes arsenite and the arsenate reductase ArsC that converts As(V) to As(III), this later being readily transported out of the cell by ArsB (Cai et al. 1998; Jackson and Dugas 2003). To date, no defined functions were assigned to the NADPH-dependent FMN reductase ArsH. It was suggested to respond to the oxidative stress caused by arsenite and recently, ArsH been demonstrated to oxidize trivalent organoarsenical herbicides to pentavalent species (Chen et al. 2015; Hervás et al. 2012). Intriguingly, since *ars* determinants are responsible for both arsenic and antimony (Sb) resistance (Branco et al. 2008; Cai et al. 1998; Carlin et al. 1995), strain S13.1.2 was tested for resistance to Sb(III) salt and found to be sensitive. This may imply a single substrate specificity of *P*. *putida* strain S13.1.2 ArsR protein towards arsenic and it is also possible that presence of As(III) is required to confer resistance to Sb(III).

As concluding remarks, our findings has demonstrated the versatility and adaptation of *P*. *putida* strain S13.1.2 to the grapevine, copper rich soils and towards the persistent effect of the soil contamination by some heavy metals on the resident microbiota. The comprehensive profiling of heavy metal resistance features also demonstrated this strain as potential tool for bioremediation procedures on vineyard or crop related soils.

## Additional file

10.1186/s13568-016-0269-x Additional figures and table.

## Abbreviations

Ag: silver
As: arsenic
Cs: cesium
Cd: cadmium
Cl: chloride
Co: cobalt
Cu: copper
K: potassium
Na: sodium
Ni: nickel
Sb: antimony
Zn: zinc
RND: resistance-nodulation-cell division
OMF: outer membrane factors
