# Supplementary material for: Comprehensive genomic and phenotypic metal resistance profile of Pseudomonas putida strain S13.1.2 isolated from a vineyard soil
Source: AMB Express. 2016 Oct 12;6:95. doi: 10.1186/s13568-016-0269-x (PMC5059233; doi:10.1186/s13568-016-0269-x)
Supplement: Supplementary file 1 — 10.1186/s13568-016-0269-x Additional figures and table. [file 13568_2016_269_MOESM1_ESM.pdf]

Comprehensive genomic and phenotypic metal resistance profile of *Pseudomonas putida* strain S13.1.2 isolated from a vineyard soil

Teik Min Chong[1], Wai-Fong Yin[1], Jian-Woon Chen[1], Samuel Mondy[2], Catherine Grandclément[2], Denis Faure[2], Yves Dessaux[2] and Kok-Gan Chan[1*]

[*]Corresponding author: Institute of Biological Sciences, Faculty of Science, University of Malaya, 50603 Kuala Lumpur, Malaysia. Tel: +60162868066. E-mail: kokgan@um.edu.my

[1] Division of Genetics and Molecular Biology, Institute of Biological Sciences, Faculty of Science, University of Malaya, 50603 Kuala Lumpur, Malaysia [2] Institute for Integrative Biology of the Cell (I2BC), CEA, CNRS, Université Paris-Sud, Université Paris-Saclay, 91198 Gif-sur-Yvette, France.

Teik Min Chong (teikminchong@um.edu.my)

Wai-Fong Yin (yinwaifong@yahoo.com)

Jian-Woon Chen (cjw246@hotmail.com)

Samuel Mondy (samuel.mondy@orange.fr)

Catherine Grandclément (catherine.grandclement@isv.cnrs-gif.fr)

Denis Faure (denis.faure@i2bc.paris-saclay.fr)

Yves Dessaux (yves.dessaux@i2bc.paris-saclay.fr)

*Kok-Gan Chan (kokgan@um.edu.my)

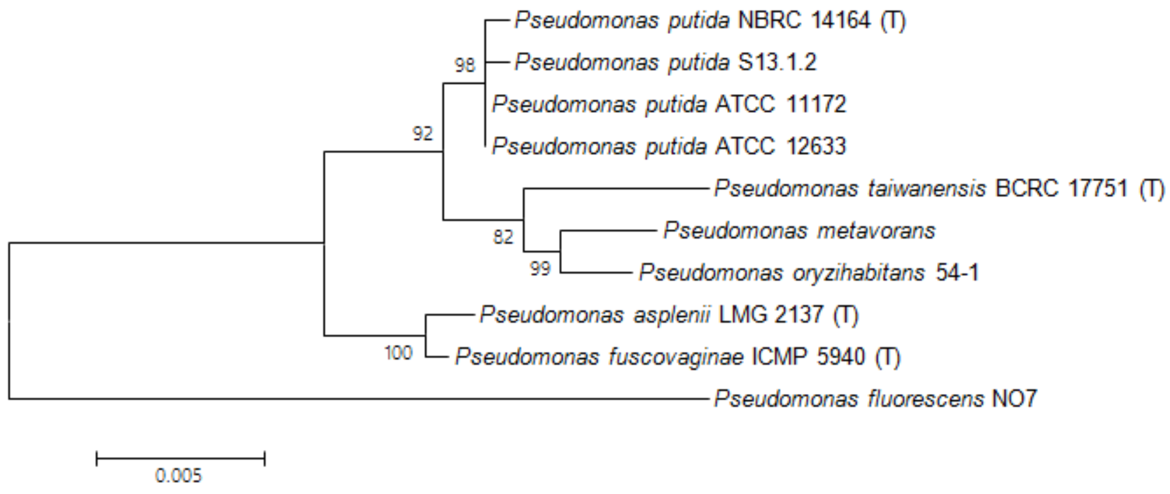

Figure S1. Phylogenetic tree highlighting the position of *P. putida* strain S13.1.2 relative to other type and non-type strains within the *Pseudomonas* genus. The strains and their corresponding GenBank accession numbers for 16S rRNA genes are (type=T): *P. putida* strain NBRC14164T, AP013070.1; *P. putida* strain S13.1.2, CP010979; *P. putida* strain ATCC11172, AF094745; *P. putida* strain ATCC12633, D37923.1; *P. taiwanensis* strain BCRC17751T, EU103629.2; and *P. metavorans*, AB302395; *P. oryzihabitans* strain 54-1, AB675634.1; *P. asplenii* strain LMG2137T, AB021397.1 and *P. fuscovaginae* strain ICMP5940T, FJ483519.1. Molecular Evolutionary Genetic Analysis (MEGA) version 6 was employed for the phylogenetic analysis incorporating Maximum Likelihood method based on Jones-Taylor-Thornton (JTT) model. The percentage of each associated taxa clustered using bootstrap test of 1000 replicates is illustrated next to the branches. The scale bar represents 0.005 substitutions per nucleotide position. *P. fluorescens* strain NO7 (FJ972536.1) was used as outgroup.

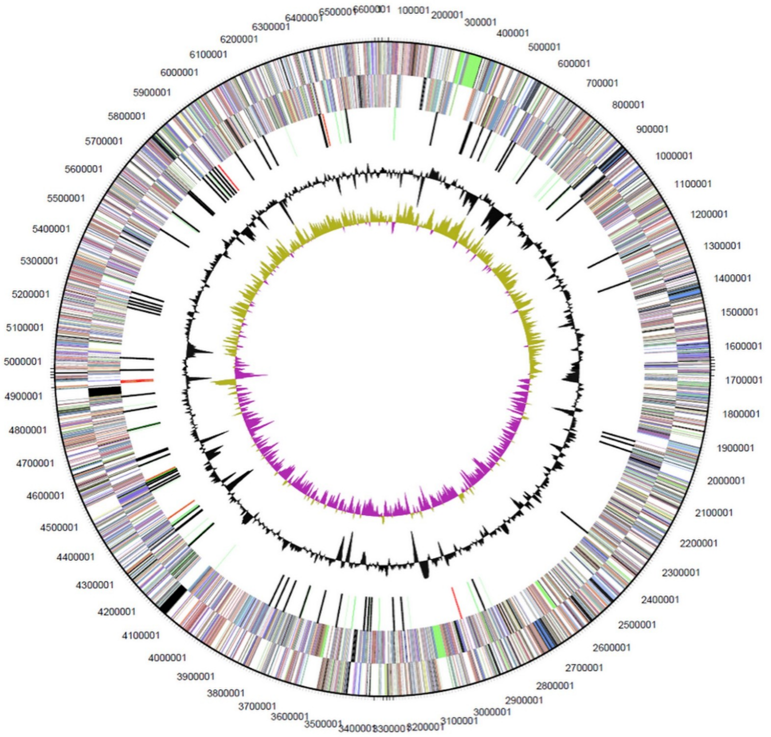

Color code of function category for top COG hit is shown below.

COG Code	COG Function Definition
[A]	RNA processing and modification
[B]	Chromatin structure and dynamics
[C]	Energy production and conversion
[D]	Cell cycle control, cell division, chromosome partitioning
[E]	Amino acid transport and metabolism
[F]	Nucleotide transport and metabolism
[G]	Carbohydrate transport and metabolism
[H]	Coenzyme transport and metabolism
[I]	Lipid transport and metabolism
[J]	Translation, ribosomal structure and biogenesis
[K]	Transcription
[L]	Replication, recombination and repair
[M]	Cell wall/membrane/envelope biogenesis
[N]	Cell motility
[O]	Posttranslational modification, protein turnover, chaperones
[P]	Inorganic ion transport and metabolism
[Q]	Secondary metabolites biosynthesis, transport and catabolism
[R]	General function prediction only
[S]	Function unknown
[T]	Signal transduction mechanisms
[U]	Intracellular trafficking, secretion, and vesicular transport
[V]	Defense mechanisms
[W]	Extracellular structures
[X]	Mobilome: prophages, transposons
[Y]	Nuclear structure
[Z]	Cytoskeleton
[NA]	Not Assigned

Figure S2. Graphical map of *P. putida* strain S13.1.2 complete chromosome. From outside to center: Genes on forward strand (colors categorized by COG), genes on reversed strand (colors categorized by COG), RNA genes (tRNAs - green; rRNAs - red; other RNAs - black), GC content, and GC skew.

Table S1: Genetic determinants of heavy metal resistance detected in the genome of strain S13.1.2 that correspond to phenotypes displayed on phenotype microarray analysis. Each assigned genes were verified and curated via homology search in NCBI BLAST non-redundant (nr) database.

Coding sequence (Prokka)	Annotation/ Predicted Role	ORF	Metal Resistance	Size (bp)	Position (Start-Stop)	Orientation
Prokka0035	Copper resistance protein B	*copB1*	Cu^{2+}	1,116	38088-36973	←
Prokka0036	Multicopper oxidase	*mco*		324	38401-38078	←
Prokka0037	Copper resistance protein A	*copA1*		1,914	40329-38416	←
Prokka0039	Transcriptional activator protein	*copR1*		678	40883-41560	→
Prokka0040	Sensor kinase	*copS*		1,377	41560-42936	→
Prokka0041	Transcriptional repressor	*frmR*		276	43053-43328	→
Prokka0043	Outer membrane efflux protein	*tolC*		1,251	44850-46100	→
Prokka0044	Cation efflux system protein	*cusB*		1,464	46097-47560	→
Prokka0045	Cation efflux system protein	*cusA1*		1,785	47557-49341	→
Prokka0046	Cation efflux system protein	*cusA2*		1,356	49359-50714	→
Prokka0047	Copper binding periplasmic protein	*cusF*		354	50711-51064	→
Prokka1751	Copper-importing P-type ATPase A	*copA2*		2,475	1823028-1820554	←
Prokka4547	Transcriptional activator protein/ copper sensing two component system response regulator	*copR2/ cusR*		681	4730050-4730730	→
Prokka4548	Sensor kinase	*cusS*		1,353	4730727-4732079	→
Prokka5227	Multicopper oxidase	*mco*		1,383	5445588-5444206	←
Prokka5685	Copper chaperone	*copZ*		198	5917384-	←

					5917187	
Prokka5687	Copper-exporting P-type ATPase A	*copA3*		2,400	5917968-5920367	→
Prokka5688	HTH-type transcriptional regulator /Cu(I) responsive transcription regulator	*hmrR*		411	5920364-5920774	→
Prokka2199	Nickel/cobalt homeostasis protein precursor	*rcnB*	Ni^{2+}	291	2333070-2332780	←
Prokka3649	Nickel transport operon	*nikR*		426	3763101-3762676	←
Prokka3650		*nikA*		1466	3763297-3764762	→
Prokka3652		*nikB*		942	3764764-3765705	→
Prokka3653		*nikC*		846	3765702-3766547	→
Prokka3654		*nikD*		771	3766548-3767318	→
Prokka3655		*nikE*		885	3767315-3768199	→
Prokka0042	Cadmium, cobalt and zinc/H(+)-K(+) antiporter	*czcD*	Co^{2+}, Zn^{2+}, Cd^{2+}	939	43336-44274	→
Prokka4214	Cobalt-zinc-cadmium resistance protein	*czcA*		3,147	4359161-4356015	←
Prokka4215		*czcB*		1,215	4360392-4359178	←
Prokka4216		*czcC*		1,254	4361638-4360385	←
Prokka3880	HTH-type transcriptional repressor	*arsR*	As^{3+}, As^{5+}	348	4000705-4001052	→
Prokka3881	Arsenical pump membrane protein	*arsB1*		1,284	4001074-4002357	→
Prokka3882	Arsenate reductase	*arsC1*		471	4002385-4002855	→
Prokka3883	NADPH-dependent FMN reductase	*arsH*		702	4002868-4003569	→
Prokka4375	Arsenical pump membrane protein	*arsB2*		420	4528908-4529327	→
Prokka4376	Arsenical pump membrane	*arsB3*		918	4529273-	→

	protein				4530190	
Prokka5064	Arsenate reductase	*arsC2*		354	5282767-<br>5283120	→
